# Dancing with the enemy: symbiotic relationships between plant RNA viruses and their hosts

**DOI:** 10.3389/fpls.2025.1716996

**Published:** 2025-11-27

**Authors:** Abhisha Roy, Bipasha Bhattacharjee, Vipin Hallan

**Affiliations:** 1 Plant Virology Lab, Council of Scientific and Industrial Research (CSIR)-Institute of Himalayan Bioresource Technology (IHBT), Palampur, Himachal Pradesh, India; 2 Academy of Scientific and Innovative Research (AcSIR), Ghaziabad, Uttar Pradesh, India; 3 Laboratory of Cell Cycles of Algae, Centre Algatech, Institute of Microbiology of the Czech Academy of Sciences, Třeboň, Czechia

**Keywords:** RNA viruses, symbiotic relationship, immunity, plant adaptation, agriculture

## Abstract

While many plant viruses cause diseases that reduce crop yield, quality, and overall plant health, not all viruses are purely detrimental. Under certain conditions, some can confer beneficial effects, including improving abiotic stress tolerance, enhancing immunity, or even increasing pollination efficiency. RNA viruses, though most often associated with disease, can also establish symbiotic relationships with their hosts that are mutualistic, commensal, or conditionally beneficial depending on environmental factors. This mini-review summarizes how mild viral infections can protect plants against more severe pathogens (cross-protection), induce signaling and epigenetic changes that enhance stress tolerance, and serve as tools for gene delivery and crop improvement. Collectively, these findings underscore the potential of RNA viruses to support plant adaptation and survival, offering innovative possibilities for sustainable agriculture and climate resilience.

## Graphical Abstract

**Figure:**
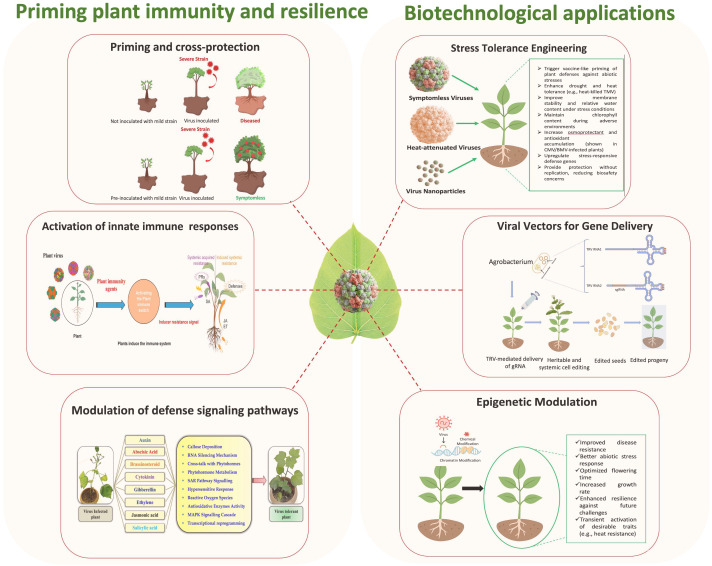
Mutualistic roles of plant RNA viruses in priming plant immunity and advancing biotechnological applications. Beyond their pathogenic reputation, RNA viruses can enhance plant resilience by priming immune responses, conferring cross-protection, and modulating defense signaling pathways. At the same time, biotechnological applications leverage viral symbiosis for stress tolerance engineering, gene delivery vectors, and epigenetic modulation of traits. Together, these interactions highlight the potential of viruses as ecological partners and innovative tools for sustainable crop improvement.

## 1 Introduction

RNA viruses are widely recognized as agents of disease in animals and plants. In humans and livestock, influenza viruses cause seasonal epidemics, rabies virus induces lethal encephalitis, and foot-and-mouth disease virus devastates livestock herds (Woolhouse et al., 2013; Villa et al., 2021). In plants, RNA viruses such as tobacco mosaic virus (TMV) affect tobacco, tomatoes, and peppers, causing mottling, leaf distortion, and stunted growth (Prabahar et al., 2015). Potato virus Y (PVY) infects potatoes, peppers, and tomatoes, reducing tuber quality (Scholthof et al., 2011). Rice tungro virus threatens rice cultivation in Southeast Asia (Tyagi et al., 2008), and cucumber mosaic virus (CMV) infects over 1,200 species, causing leaf mosaics and malformed fruits while spreading efficiently via aphid vectors (Ando et al., 2019; Rattan et al., 2022).

However, not all plant-virus interactions are harmful. Recent studies reveal that many RNA viruses can exist without causing disease and may confer benefits, including immune system conditioning, stress resilience, competitive advantages, evolutionary adaptability, and facilitation of symbiotic partnerships. Cucumber mosaic virus (CMV) is a plant RNA virus that often persists asymptomatically in its host plants. Studies have shown that CMV infection can enhance plant resilience to abiotic stresses such as drought and freezing by increasing levels of osmoprotectants and antioxidants (Xu et al., 2008). This suggests that CMV can function as a mutualistic partner under specific environmental conditions, contributing to the plant’s survival and adaptation. In yeast, killer viruses produce toxins that eliminate competing strains, protecting host populations (Schmitt and Breinig, 2002). Viral genetic material can also drive evolutionary innovation by influencing host gene regulation or integrating into host genomes (Sanjuán and Domingo-Calap, 2016; Broecker and Moelling, 2019). In plants, RNA viruses can subtly modulate physiology by altering hormone and stress signaling or small RNA pathways, sometimes enhancing growth, defense, or reproduction. For example, CMV infection alters floral scent to attract bumblebees, increasing pollination and seed set despite pathogenic effects (Groen et al., 2016). Furthermore, RNA viruses have been repurposed for gene delivery and therapeutic applications (Zhu et al., 2023; Lundstrom, 2023).

In this mini review, we explore how RNA viruses can function as benefactors of their plant hosts. We discuss several facets of this phenomenon: (1) Pathogens turned protectors, where viral infections boost plant immunity or protect against other diseases; (2) Mutualistic viruses enhancing stress tolerance and their emerging applications in agriculture; (3) the use of mild RNA viruses as tools for gene delivery and epigenetic modulation of traits; and finally (4) the challenges and future prospects of harnessing viral symbioses in plant science and biotechnology. By examining recent findings and longstanding examples, we aim to provide a comprehensive view of the “bright side” of plant RNA viruses and outline how these insights can be translated into innovative strategies for crop improvement and sustainable agriculture.

## 2 Pathogens turned protectors: the defense-boosting side of RNA viruses

Despite their notorious reputation for spreading disease, certain RNA viruses have the paradoxical ability to shield their hosts from other infections. Complex immune systems have developed in plants, and some viral infections activate these defenses in ways that make the plant more resilient to future assaults. Here, we go over some defense-enhancing strategies that can turn an “enemy” virus into the plant’s protector.

### 2.1 Priming and cross-protection

Plants (or other hosts) can be infected by a mild or attenuated strain of a virus, which can then prevent or lessen the effects of a subsequent infection by a more virulent strain of the same or a related virus. Competition for resources, cellular site occupancy, or immune system activation are the causes of this. In citrus horticulture, for instance, weak strains of the Citrus tristeza virus (CTV) are used to shield trees from more severe strains; this technique is called mild strain cross-protection (MSCP). By inoculating citrus plants with a mild CTV isolate, this method can stop or lessen the impact of later infections by more virulent strains (Lee and Keremane, 2013; Licciardello et al., 2023). ​Similar protection has been achieved in case of Sugarcane mosaic virus (SCMV) (Xu et al., 2021), cucumber mosaic virus (CMV) (Zhou and Zhou, 2012), papaya ringspot virus (PRSV) (Resour et al., 2025) etc. By identifying or generating hypovirulent mutant strains, which are virus variants with reduced virulence that cause mild or no disease symptoms, researchers have explored their potential to trigger protective responses in host plants. Similar strategies have been applied to other crop-virus systems, such as papaya ringspot virus in papaya and sugarcane mosaic virus in sugarcane. In terms of mechanism, cross-protection frequently depends on the host’s RNA silencing module: the mild virus generates replicative intermediaries of double-stranded RNA that cause gene silencing, thereby targeting the genomes of any inbound related virus. In essence, the plant’s antiviral defense (particularly small interfering RNAs) gets primed by the mild strain, so that a subsequent invasion by a related virus is rapidly suppressed. This was demonstrated in studies of TMV and other viruses, where pre-infection induced virus-specific small RNAs and other defense responses that correlated with resistance to a challenge inoculation (Voloudakis et al., 2025). Intriguingly, there are instances of heterologous protection-a mild virus defending against another virus-which may arise from widespread immune stimulation, but cross-protection is typically virus-strain-specific. All things considered, viral priming and cross-protection show a definite mutualistic result: the host is protected from lethal virus strains (and possibly other infections) while the virus obtains a viable host in which to reproduce. In the agricultural sector, this approach can be applied by using mild variants of pepino mosaic virus to protect greenhouse tomatoes from more severe strains, thus offering a chemical-free disease control strategy compatible with sustainable crop production (Khankhum et al., 2016).

### 2.2 Activation of innate immune responses

In addition to intentional cross-protection techniques, a plant may become more defensive even after an unintentional viral infection. The generic features of viruses, such as double-stranded RNA (dsRNA) produced during replication as a “non-self” molecular pattern, can be recognized by pattern-recognition receptors (PRRs) in plant cells, leading to their activation (Hur, 2019). This triggers the plant’s innate immune responses, leading to the production of defense proteins, RNA-silencing enzymes, and antiviral compounds. These responses not only help combat the current pathogen but also prime the plant’s defenses more broadly, enhancing resistance against future infections. A mild virus infection, for instance, may result in the systemic buildup of pathogenesis-related (PR) proteins, increased antioxidant levels, or reinforced cell walls, all of which may unintentionally reduce the plant’s susceptibility to unrelated diseases like bacteria or fungus. Thus, in addition to fighting the current infection, RNA viruses prime the plant’s immune system thus making it possible for the plant to react more robustly to later attacks by various diseases, such as fungus and bacteria (Pumplin and Voinnet, 2013; Leonetti et al., 2021). According to research, some viruses trigger the immune response by boosting salicylic acid or other defense hormones, which in turn cross-activates pathways that provide resistance against recurrent infections. In one instance, a common bean’s latent endornavirus infection was linked to increased basal expression of defensive genes, which enabled the plants to withstand a subsequent challenge from a pathogenic virus (Khankhum et al., 2016). The net impact, however, may be a sort of immune “alert” status that helps the plant in the event of additional attack when a virus causes moderate immune responses without leading to severe disease.

### 2.3 Modulation of defense signaling pathways

Upon infection, RNA viruses can interfere with the plant’s immune signaling to avoid detection and suppression. While these action aim to facilitate viral replication, they can also lead to a state of heightened alertness in the plant’s immune system (Ellendorff et al., 2009). For example, it has been demonstrated that Turnip mosaic virus (TuMV) infection increases the expression of genes involved in the abscisic acid (ABA) signaling pathway. The virus suppresses the plant’s immune response to facilitate infection. However, the mild activation of ABA can prime the plant to tolerate other abiotic stresses (like drought or salinity), leading to an unintended increase in stress tolerance (Shukla et al., 2025). The virus’s modification of hormonal crosstalk results in a “unintended increase in stress tolerance,” according to Shukla et al. (2025). In other situations, viruses that increase salicylic acid to evade detection may also increase the plant’s resistance to other diseases, as salicylic acid is an important signal for defense against bacteria and biotrophic fungi. Conversely, a virus that interferes with jasmonate signaling, possibly to increase the plant’s appeal to insect vectors, may unintentionally decrease herbivory by non-vector insects, which would be advantageous to the host. Although these outcomes are context-dependent and not fully understood, they illustrate that viral manipulation of host immunity can occasionally produce conditionally beneficial effects, highlighting a nuanced spectrum in virus-host interactions where primarily harmful infections may confer incidental advantages under specific environmental or stress conditions.

## 3 Exploring the role of mutualistic RNA viruses in enhancing crop resilience and biotech applications

### 3.1 Stress tolerance engineering

Emerging research highlights the remarkable potential of viruses in enhancing plant stress tolerance. A striking example comes from the tripartite symbiosis described by (Urayama et al., 2024), where panic grass (*Dichanthelium lanuginosum*), its fungal endophyte (*Curvularia protuberata*), and the associated Curvularia thermal tolerance virus (CThTV) enable survival in geothermal soils with temperatures reaching 65°C. Extending this principle, inoculation of rice and t2023omato with similar fungus-virus combinations has significantly improved heat tolerance (Al-Hamdani et al., 2015). Inspired by such natural partnerships, biotechnologists are now exploring engineered viral symbionts to fortify crops against climate change. For instance, modified Tobacco mosaic virus (TMV) has been used as a carrier to prime immune responses and enhance drought resistance without causing disease (Mandadi and Scholthof, 2013), while TMV nanoparticles mitigate oxidative stress under water-limited conditions (Al-Khayri et al., 2023). Similarly, persistent, symptomless viruses such as endornaviruses in beans and squash are being investigated as potential tools for strengthening crop performance (Uchida et al., 2021). Parallel efforts are also focused on discovering and utilizing naturally occurring, non-symptomatic viruses that improve plant resilience, such as those found in rice and cucumber (Xu et al., 2008). Plant breeders are beginning to screen crops for such beneficial infections, while synthetic biology approaches aim to repurpose viruses into safe bioengineering tools. These engineered viruses can deliver genes for stress tolerance, growth, and disease resistance, offering a sustainable alternative to conventional transgenic methods … Beyond replicating viruses, virus infections such as cucumber mosaic virus (CMV) and Brome mosaic virus (BMV), as well as non-replicating viral particles including heat-killed Tobacco mosaic virus (TMV), have been shown to trigger protective, “vaccine-like” responses against abiotic stresses (Tomitaka et al. 2024). For example, Xu et al. (2008) demonstrated that CMV or BMV infections delayed drought-symptom onset and increased osmoprotectant and antioxidant levels, providing evidence of virus-mediated abiotic stress resilience. More recently, Augustine et al. (2023) showed that treatment of *Nicotiana tabacum* with heat-killed TMV enhanced drought and heat tolerance by improving membrane stability, relative water content, chlorophyll content, and upregulating stress-responsive genes. Together, these findings suggest that viruses, long considered only as pathogens, may serve as “viral probiotics,” offering farmers novel, eco-friendly means to enhance stress tolerance in crops. While further research is needed to ensure biosafety and long-term stability, the growing body of evidence positions viruses not merely as threats but as promising allies in sustainable agriculture. By harnessing both natural viral partnerships and engineered viral tools, we may unlock innovative strategies to help crops withstand the increasing challenges of climate change.

### 3.2 Viral vectors for gene delivery

Viral vectors are emerging as efficient tools for plant gene delivery, offering rapid and systemic expression of beneficial traits. Symbiotic or persistent RNA viruses, which naturally coexist with plants without causing disease, are being engineered to deliver genes for stress tolerance, growth, and disease resistance. Their ability to spread through plant tissues provides a sustainable alternative to conventional transgenic methods. Engineered Tobacco mosaic virus (TMV) particles and attenuated forms of Alfalfa mosaic virus (AMV) and Cowpea mosaic virus *(*CPMV) are now used to carry functional genes and gene-editing tools such as CRISPR/Cas9 (Mahmood et al., 2023). Unlike traditional genetic modification, virus-mediated editing can create targeted, inheritable changes without integrating foreign DNA, reducing biosafety concerns and regulatory hurdles. Early applications of viral vectors have demonstrated tangible improvements in crop performance, such as enhanced water-use efficiency in tomato and increased resilience to drought and heat in other crops (Fan et al., 2025). Geminivirus- and tobamovirus-based vectors have been used to transiently deliver CRISPR/Cas9 components, enabling rapid functional characterization of stress-responsive genes and activation of protective pathways in multiple plant species (Yin et al., 2015; Zaidi et al., 2017, 2020; Bhattacharjee et al., 2022; Uranga et al., 2023). Additionally, Tobacco rattle virus (TRV) has been engineered to deliver guide RNAs for CRISPR/Cas9-mediated genome editing in plants. For instance, a study demonstrated that TRV-mediated delivery of guide RNAs resulted in efficient genome editing in tomato somatic cells, with mutagenesis efficiency reaching up to 65% in leaves and 50% in fruits (Wang et al., 2024). This approach allows for transient and heritable genome modifications without the need for stable transformation.

By leveraging virus-based genome-editing vectors, such as TRV and geminivirus-derived systems, this approach provides a flexible, targeted alternative to conventional breeding and transgenics, accelerating the development of resilient, high-performing crops for sustainable agriculture (Zhang et al., 2024).

### 3.3 Epigenetic modulation

Viruses can influence plant traits not only through direct infection but also via epigenetic modulation, altering gene activity without changing the DNA sequence itself. These changes are often reversible and can fine-tune traits such as flowering time, growth rate, and stress tolerance (Roossinck, 2015). A key mechanism is RNA interference (RNAi), in which viral infection perturbs small RNA pathways, including microRNAs (miRNAs) and small interfering RNAs (siRNAs), which play critical roles in regulating plant development and defense. For instance, cucumber mosaic virus (CMV) infection in Arabidopsis was shown to disrupt small RNA balances, shifting flowering regulation and stress responses (Takahashi et al., 2022). Similarly, viral infections can reshape DNA methylation and histone modification patterns, activating or silencing stress-responsive genes (Roossinck, 2011). In some cases, these modifications leave a “stress memory,” priming plants for enhanced resilience against future challenges (Xu et al., 2008; Bhar et al., 2021). Beyond natural infections, this opens exciting biotechnological possibilities. Because viruses can act as temporary switches, researchers have explored deploying engineered viral systems or RNA-based sprays to transiently activate desirable traits (Gentzel et al., 2022). For example, foliar application of double-stranded RNA (dsRNA) has been shown to transiently silence specific genes in plants, enabling the activation or deactivation of traits without permanent genetic modification (McRae et al., 2023). Similarly, high-pressure spraying of dsRNAs can trigger the plant RNA interference (RNAi) machinery, leading to transient gene silencing and enhanced disease resistance (Nerva et al., 2022). Virus-induced gene silencing (VIGS) systems exploit antiviral defense pathways to suppress targeted genes transiently, providing another strategy for reversible trait modulation (Zulfiqar et al., 2023). Beyond RNAi, small interfering RNAs (siRNAs) delivered via viral vectors can guide RNA-directed DNA methylation (RdDM), inducing reversible epigenetic changes that allow plants to dynamically adapt to environmental stresses (Wagh et al., 2025; Papareddy et al., 2020). More recently, synthetic-tasiRNA-based VIGS (syn-tasiR-VIGS) systems have demonstrated that engineered viral vectors can deliver small RNAs to transiently modulate gene expression, highlighting the potential of viruses as tools for dynamic epigenetic reprogramming (Cisneros et al., 2025). Collectively, these approaches illustrate a paradigm shift in which viruses are not merely pathogens but can be harnessed to provide crops with timely, reversible advantages, offering innovative avenues for climate-smart, sustainable agriculture.

## 4 Where there’s a boon, there’s a bane

Despite their potential benefits, the use of RNA viruses in plants is not without significant limitations and risks. RNA viruses are inherently prone to high mutation rates and genetic recombination, which can generate novel strains with altered host ranges or pathogenicity, potentially leading to unintended outbreaks (Sanjuán and Domingo-Calap, 2016; Woolhouse et al., 2013). Cross-species transmission is another concern, as viruses that are non-pathogenic in one host may become pathogenic when infecting related or unrelated plant species, posing ecological and agricultural risks (Scholthof et al., 2011; Roossinck, 2015). Engineered or mild strains used for cross-protection or gene delivery could also recombine with wild-type viruses, creating virulent hybrids that threaten crops (Lee and Keremane, 2013; Pechinger et al., 2019). Moreover, RNA viruses can interfere with native plant microbiomes or trigger unintended epigenetic changes that may affect growth or stress responses (Papareddy et al., 2020; Zhu et al., 2023). Environmental factors, vector dynamics, and host susceptibility further complicate their deployment in agriculture, making field applications unpredictable.

**Figure 1:**
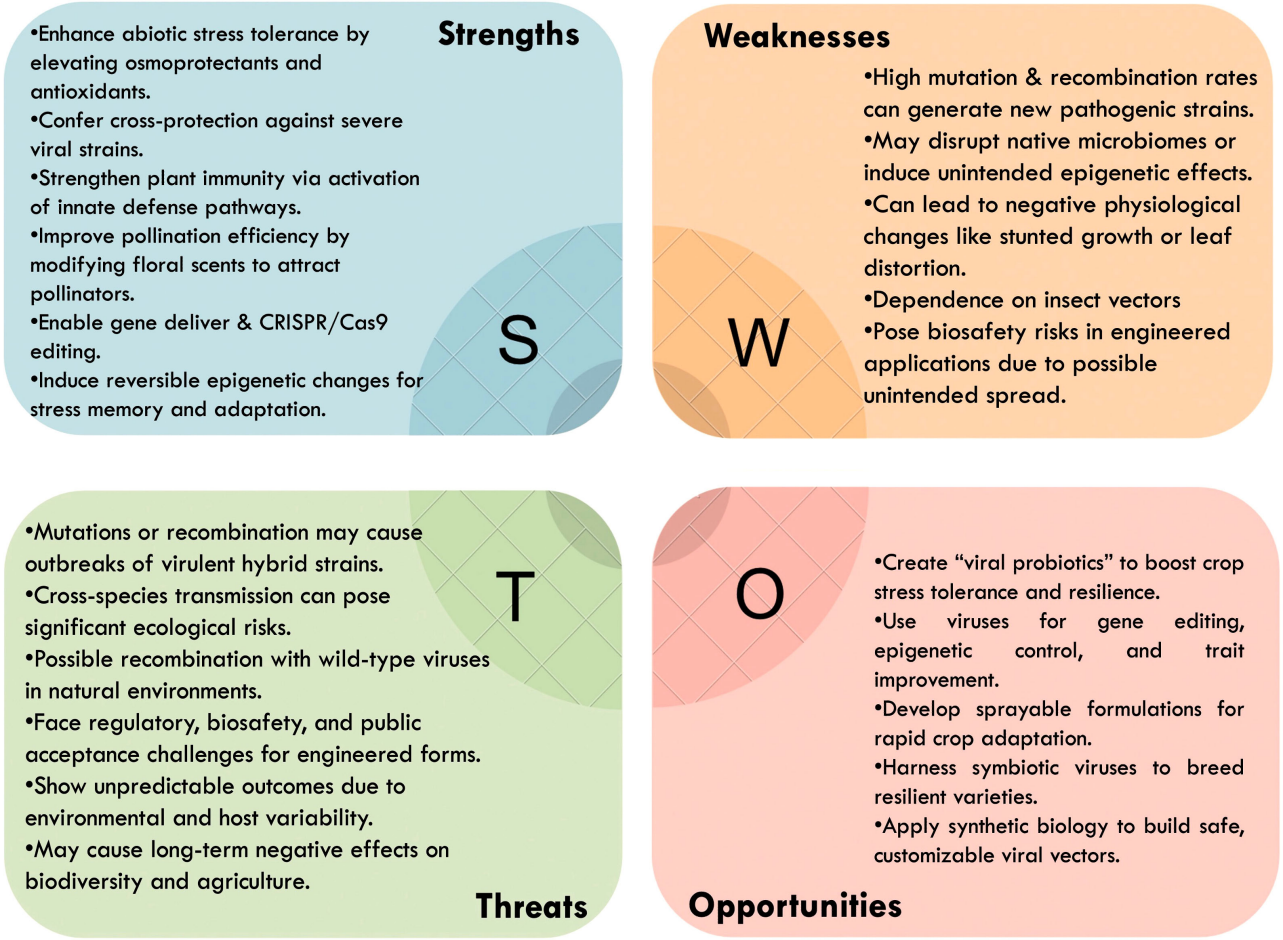
SWOT analysis diagram illustrating the strengths, weaknesses, opportunities, and threats associated with the use of plant RNA viruses in agriculture, highlighting their potential as mutualistic agents for stress tolerance and biotechnological applications, alongside the inherent risks and challenges.

A deeper understanding of host-virus-environment interactions is needed to ensure consistency and predictability of beneficial effects. Long-term ecological and biosafety assessments will be critical to mitigate risks such as unintended viral recombination or cross-species transmission. While advances in synthetic biology, nanotechnology, and genome editing offer tools to develop customized viral vectors, their successful deployment will require careful regulatory frameworks and acceptance by farmers and stakeholders. To summarize the multifaceted considerations, a SWOT analysis illustrating the Strengths, Weaknesses, Opportunities, and Threats associated with the use of plant RNA viruses in agriculture has been included and is presented in **Figure 1**. Therefore, while RNA viruses hold promise as mutualistic partners and biotechnological tools, these inherent challenges must be addressed before practical application in agriculture.

## 5 Future perspectives

The emerging evidence surrounding mutualistic RNA viruses highlights a paradigm shift in plant virology, from perceiving viruses solely as destructive pathogens to recognizing them as potential allies in agriculture. Across stress tolerance engineering, viral vectors for gene delivery, and epigenetic modulation, it is increasingly evident that viruses can be strategically harnessed to enhance resilience, adaptability, and productivity in crops. Looking ahead, the use of mutualistic viruses as “viral probiotics,” bio-safe gene delivery agents, and epigenetic modulators opens a new frontier in crop biotechnology. Viral epigenetic modulation, in particular, offers the exciting possibility of transient, reversible trait enhancement without permanent genetic modification, potentially easing regulatory bottlenecks and public concerns surrounding GMOs. Moreover, the concept of field-deployable viral sprays or formulations that temporarily enhance resilience could redefine crop management practices, allowing crops to be “tuned” to environmental challenges in real time. By bridging natural viral ecology with engineered applications, future research may transform these hidden allies into practical tools for building climate-resilient and food-secure cropping systems.

In conclusion, mutualistic RNA viruses represent an untapped resource in sustainable agriculture, combining ecological adaptability with cutting-edge biotechnology. By bridging natural viral ecology with engineered applications, future research may transform these hidden allies into practical tools for building climate-resilient and food-secure cropping systems.
